# Cellular Targets of Nitric Oxide in the Hippocampus

**DOI:** 10.1371/journal.pone.0057292

**Published:** 2013-02-25

**Authors:** Katalin Bartus, Beatrice Pigott, John Garthwaite

**Affiliations:** The Wolfson Institute for Biomedical Research, University College London, London, United Kingdom; Max Planck Institute of Psychiatry, Germany

## Abstract

In the hippocampus, as in many other CNS areas, nitric oxide (NO) participates in synaptic plasticity, manifested as changes in pre- and/or postsynaptic function. While it is known that these changes are brought about by cGMP following activation of guanylyl cyclase-coupled NO receptors attempts to locate cGMP by immunocytochemistry in hippocampal slices in response to NO have failed to detect the cGMP elevation where expected, i.e. in the pyramidal neurones. Instead, astrocytes, unidentified varicose fibres and GABA-ergic nerve terminals are reported to be the prominent NO targets, raising the possibility that NO acts indirectly via other cells. We have re-investigated the distribution of cGMP generated in response to endogenous and exogenous NO in hippocampal slices using immunohistochemistry and new conditions designed to optimise cGMP accumulation and, hence, its detectability. The conditions included use of tissue from the developing rat hippocampus, a potent inhibitor of phosphodiesterase-2, and an allosteric enhancer of the NO-receptive guanylyl cyclase. Under these conditions, cGMP was formed in response to endogenous NO and was found in a population of pyramidal cell somata in area CA3 and subiculum as well as in structures described previously. The additional presence of exogenous NO resulted in hippocampal cGMP reaching the highest level recorded for brain tissue (1700 pmol/mg protein) and in cGMP immunolabelling throughout the pyramidal cell layer. Populations of axons and interneurones were also stained. According with these results, immunohistochemistry for the common NO receptor β1-subunit indicated widespread expression. A similar staining pattern for the α1-subunit with an antibody used previously in the hippocampus and elsewhere, however, proved to be artefactual. The results indicate that the targets of NO in the hippocampus are more varied and extensive than previous evidence had suggested and, in particular, that the pyramidal neurones participating in NO-dependent synaptic plasticity are direct NO targets.

## Introduction

Nitric oxide (NO) is a freely-diffusible transmitter that is critical for the normal functioning of the nervous [1], cardiovascular [2] and immune systems [3], amongst others. In the absence of immune challenge, NO signals are generated by two NO synthase (NOS) isoforms, namely the neuronal and endothelial subtypes, abbreviated as nNOS and eNOS respectively [4]. Physiological NO signal transduction occurs via GMP, which is synthesised on activation of NO-targeted guanylyl cyclase enzymes. These proteins are the only established NO receptors and they exist in two isoforms, comprising an α1 or α2 subunit together with a common β1 subunit [5]. Hydrolysis through one or more phosphodiesterase (PDE) enzymes assists in the shaping of the resultant cellular cGMP signals [6].

In brain, NO generation from nNOS is coupled to activation of N-methyl-D-aspartate (NMDA) receptors and one of its roles is in modifying the efficacy of synaptic transmission [1], [7], such as in long-term potentiation (LTP), a persistent enhancement of synaptic strength frequently initiated by NMDA receptor channel opening, and a putative correlate of learning and memory [8]. Based on studies done primarily at hippocampal CA3/CA1 synapses, NO-dependent LTP requires phasic, activity-dependent NO formation from nNOS superimposed on a tonic low level of NO generated by eNOS, with NO in both cases acting through cGMP [9]. Interestingly, knockout studies indicate that both NO-activated guanylyl cyclase isoforms are also needed for the LTP [10].

At the cellular level, the simplest hypothesis is that NO-cGMP signalling in LTP takes place directly at the excitatory synapses between the participant pyramidal neurones, as has been suggested by evidence derived largely from dissociated hippocampal cultures [11]–[16]. When cGMP is located immunohistochemically in the intact hippocampus following exposure to exogenous NO, however, the nucleotide has been detected in astrocytes, GABAergic nerve terminals, unidentified varicose fibres, and in blood vessels, but not in pyramidal cells [17]–[26]. If correct, this distribution would suggest a more complicated scenario in which NO primarily targets cells that are not the direct participants in LTP.

In an attempt to resolve the apparent mismatch between the functional and anatomical evidence, the present work re-examines the location of NO-stimulated cGMP accumulation in hippocampal slices using previously untested experimental conditions designed to maximise the cGMP response and thereby reveal targets of NO that may have been below the detection limit beforehand.

## Materials and Methods

### Ethics statement

Experiments were conducted in strict accordance with the UK Animals (Scientific Procedures) Act 1986, under a project licence from the British Home Office and with approval of the University College London ethical review panel.

### Animals

Unless otherwise stated, male, 10-day-old Sprague Dawley rats were used (Charles River, Kent, UK). Brain tissue from male, 8–19 week-old, C57/Bl6/SV129 mice lacking the NO-targeted guanylyl cyclase α1 subunit and their wild-type counterparts was kindly provided by Dr. Adrian Hobbs (University College London, London, UK). Male, 8-week-old, C57/Bl6 mice were obtained from Charles River.

### Pharmacological compounds

3-(4-Amino-5-cyclopropylpyrimidine-2-yl)-1-(2-fluorobenzyl)-1*H*-pyrazolo[3,4-b]pyridine (BAY 41-2272), 2-[(3,4-dimethoxyphenyl)methyl]-7-[(1R)-1-hydroxyethyl]-4- phenylbutyl]-5-methyl-imidazo[5,1-f][1,2,4]triazin-4(1H)-one (BAY 60-7550) and 2-(N,N-diethylamino)-diazenolate-2-oxide diethylammonium salt (DEA/NO) were purchased from Axxora (Enzo Life Sciences, Exeter, UK). 3-Isobutyl-1-methylxanthine (IBMX) and 1H-[1,2,4]oxadiazolo[4,3-a]quinoxalin-1-one (ODQ) were obtained from Sigma-Aldrich Company, (Poole, Dorset, UK) and L-nitroarginine (L-NNA) from Tocris Cookson (Avonmouth, Bristol, UK). Stock solutions were made in DMSO (BAY 41-2772, BAY 60-7550, ODQ), 10 mM NaOH (DEA/NO), equimolar HCl (L-NNA) or artificial cerebrospinal fluid (see composition below; IBMX) and were at least 100-fold more concentrated than the final concentration.

### Antisera

The primary antibodies that were used are listed in Table S1. Secondary antibodies for immunofluorohistochemistry were: donkey anti-mouse Alexa 594 (used at 1∶600), donkey anti-rabbit Alexa 594 (1∶1,500) and donkey anti-sheep Alexa 488 (1∶1,000; all from Invitrogen, Paisley, UK). A biotinylated donkey anti-rabbit secondary antibody (1∶200; Chemicon Europe, Hampshire, UK) was used for immunoperoxidase staining. Western blotting for the NO-targeted guanylyl cyclase α1 subunit or actin was done with a goat anti-rabbit (diluted 1∶15,000; Perbio Science, Northumberland, UK) or a donkey anti-goat (1∶20,000; Santa Cruz Biotechnology, Heidelberg, Germany) horseradish peroxidase-conjugated secondary antibody, respectively.

### Tissue preparation

Rats were killed by cervical dislocation and decapitation. The hippocampi were swiftly dissected out into ice-cold artificial cerebrospinal fluid comprising (in mM): 120 NaCl, 2 KCl, 1.19 MgSO_4_, 26 NaHCO_3_, 1.18 KH_2_PO_4_, 2 CaCl_2_ and 11 D-glucose, equilibrated with 95% O_2_/5% CO_2_ to pH 7.4 (at 37°C). The centre of the hippocampus was cut along the transverse plane at 400 µm intervals using a tissue chopper (McIlwain, Campden Instruments, Loughborough, UK). Slices were then left to recover in flasks of artificial cerebrospinal fluid held in a shaking water bath (37°C) and continuously perfused with 95% O_2_/5% CO_2_.

After 1–2 hr, slices were randomly distributed to new flasks of artificial cerebrospinal fluid, also held in a shaking water bath (37°C) and perfused with 95% O_2_/5% CO_2_. NO-dependent cGMP synthesis was stimulated by exposure to the allosteric enhancer of NO-targeted guanylyl cyclase BAY 41-2272 (0.3–30 µM, 5 min application) alone, or in combination with the NO donor DEA/NO (10 µM, 2 min application). To increase the cGMP signal-to-noise ratio, slices were pre-incubated for 15 min with the general PDE inhibitor IBMX (1 mM) or an inhibitor of PDE-2 (BAY 60-7550, 0.001–1 µM), the enzyme chiefly responsible for cGMP hydrolysis in the hippocampus [26], [27]. Other inhibitors were pre-applied for 25 min.

Within 30 s of the end of stimulation, a proportion of slices were submerged in 4% paraformaldehyde in 0.1 M phosphate buffer (pH 7.4 at room temperature). These tissues were subsequently used for fluorescent immunohistochemistry. At the same time the remaining slices, which were used for cGMP measurement, were individually inactivated by submersion in 250 µl boiling buffer comprising 50 mM tris-HCl and 4 mM EDTA (pH 7.4 at room temperature) for 15–20 min.

### Immunofluorohistochemistry

Slices were fixed for 2 h in 4% paraformaldehyde, washed for 60 min in 0.1 M phosphate buffer (pH 7.4 at room temperature), which was changed every 15 min, and cryoprotected using a series of sucrose solutions ascending in concentration (5–20% in 0.1 M phosphate buffer followed by 50% in optimal cutting temperature medium (Raymond A Lamb, Eastbourne, UK); all stages done at 4°C). Slices were then embedded in neat cutting medium and rapidly frozen on dry ice made extra cold with isopentane. Transverse (10 µm-thick) sections were prepared on chrome alum/gelatine-coated slides.

For immunolabelling, sections were rehydrated in tris-buffered saline containing 0.1% triton-X (TBS-T; pH 7.4 at room temperature; 2, 5 min washes) and then blocked in 10% donkey serum (Millipore, Watford, UK). After 1 h, the primary antibodies, diluted in 1% donkey serum, were applied at 4°C in a humid environment. Control sections for the selectivity of the secondary antibody received 1% serum but no primary antibody. Between 18–20 h later, sections were washed for 40 min in TBS-T (which was changed every 10 min) and incubated with the secondary antibodies for 1 h in the dark. Washes were then repeated and sections mounted in Vectashield medium containing the nuclear counterstain 4′,6′-diamidino-2-phenylindole (DAPI; both Vector Laboratories, Peterborough, UK). Unless otherwise stated, solutions were prepared in TBS-T and applied at room-temperature. Z-stacks were captured using a Leica TCS SP confocal microscope running Leica LCS SP2 software (Leica Microsystems, Heidelberg, Germany).

### cGMP measurement

cGMP was measured by radioimmunoassay and normalised to the total slice protein determined using the bicinchoninic acid method (Perbio Science).

### Immunoperoxidase staining

Rats were deeply anaesthetised using sodium pentobarbitone (200 mg/ml/animal, injected intraperitoneally) and intracardially perfused with ice-cold 0.1 M phosphate buffered saline, followed by 1% cold paraformaldehyde (prepared in 0.1 M phosphate buffer). The hippocampi were removed and fixed for a further 3 h in 1% paraformaldehyde. Mice were killed by cervical dislocation and decapitation. Mouse brains (minus cerebella, see western blotting) were submerged in ice-cold paraformaldehyde (4%, unless otherwise stated). The anterior and posterior ends of the brain were trimmed, leaving the middle of the hippocampi intact, and the hemispheres were separated and fixed for a further 2 h at 4°C. All of the tissues were cryoprotected, sectioned and then rehydrated as for immunofluorohistochemistry. Sections were stained according to published methods [28], except that the peroxidase suppressor (Perbio Science) was applied for 15 min prior to blocking and that rat tissues were permeabilised with neat acetone for 5 min immediately after rehydration. Counterstaining was done with Mayer's hemalum (Merck, Sharpe and Dohme, Hertfordshire, UK). Control sections for the selectivity of the secondary antibody did not receive the primary antibody.

### Western blotting

The cerebella of the wild-type and NO-targeted guanylyl cyclase α1-knockout mice that were used for immunoperoxidase staining were removed and homogenised in an ice-cold tissue-lysis buffer containing a protease inhibitor cocktail (Halt, Fisher Scientific, Leicestershire, UK) and 1% sodium dodecyl sulphate. The homogenate was heated (70°C for 10 min) and centrifuged at 13,000 rpm for 10 min (5°C). 50 µg of protein (measured using the bicinchoninic acid method) in the resulting supernatant were then separated by sodium dodecyl sulphate polyacrylamide gel electrophoresis and transferred to a polyvinylidene fluoride membrane. The gel running buffer comprised (in mM): 25 tris base, 192 glycine and 3.5 sodium dodecyl sulphate. The gel to membrane transfer buffer additionally contained 2.5% methanol. Membranes were then rinsed in double distilled H_2_O and then tris-buffered saline (TBS; pH 7.4 at room temperature), blocked for 1 h in TBS containing 3% skimmed milk and washed for 5 min in TBS. The NO-targeted guanylyl cyclase α1 primary antibody was then applied for 1 h, and the membranes were washed (3, 5 min washes in TBS containing 0.05% tween-20, then 1, 5 min wash in TBS) and exposed to a horseradish peroxidase-conjugated secondary antibody. Antibodies were diluted in 50% blocking buffer. After 1 h, washes including TBS/tween-20 were repeated and antibody binding was imaged on chemiluminescent film using the SuperSignal West Pico Chemiluminescent Substrate system (Thermo Fischer, Leicestershire, UK). To control for effective protein loading, the membranes were then stripped of the α1 antibody using Thermo Fischer Restore Western Blot Stripping Buffer, washed (TBS, 5 min) and probed for actin by the above methods, but incorporating extra washes to prevent background staining and overexposure of the film.

### Analysis

For histology, photographs are representative of data collected from at least 3 animals (2 knock-out animals). Experimental and control sections were prepared, stained and photographed in parallel. The experimenter was blinded to the genotype of wild-type and knock-out sections until after photographs were taken. Control and experimental images were adjusted equally for brightness and contrast using Adobe Photoshop (Adobe, California, USA).

cGMP measurements are mean values ± standard error of the mean and were collected using slices from at least 3 rats. Statistical significance was determined by analysis of variance (ANOVA) with Dunnett's *post hoc* test using GraphPad InStat 3 software (GraphPad Software Inc., California, USA) and was concluded when *p*<0.05.

For immunoblot analysis, films were scanned and the average grey value across each horizontal row of pixels in each vertical protein lane was calculated using ImageJ (National Institutes of Health Science, Maryland, USA). Lysates obtained from wild-type and knock-out mice were processed on the same gels/membranes and scanned images were adjusted equally for brightness and contrast using Microsoft PowerPoint (USA). Data shown were collected using cerebellar lysates and are representative of forebrain lysates.

## Results

### Immunofluorescence for NO-evoked cGMP accumulation

Attempts to locate NO-evoked cGMP accumulation were carried out using immature rat hippocampus. The young tissue provides superior preservation during in vitro incubation [29] and higher amplitude NO-dependent cGMP responses to NMDA [30] compared with tissue from the adult.

**Figure 1:**
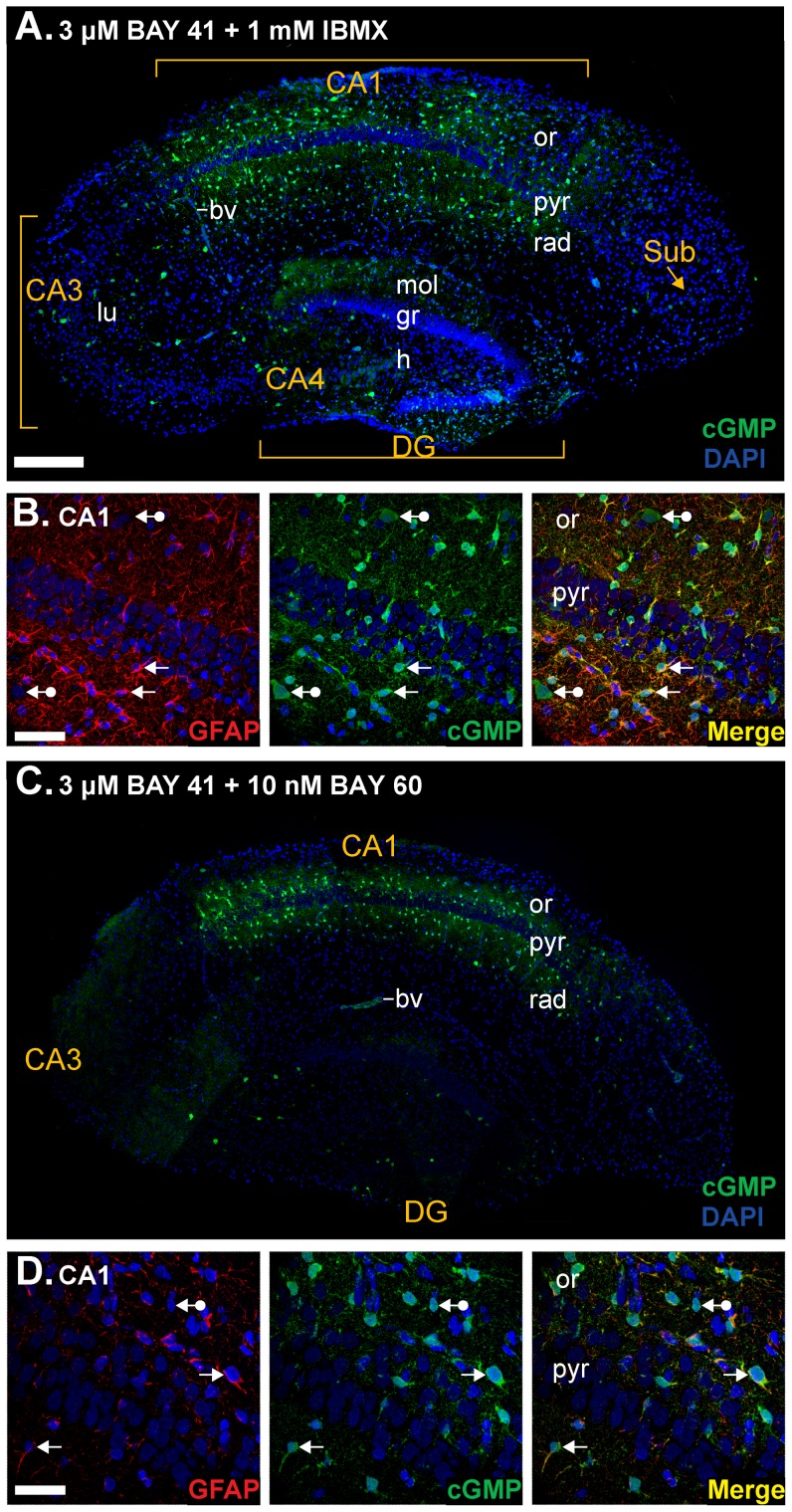
cGMP immunohistochemistry in immature rat hippocampal slices. **A,B.** Sections from slices pre-treated with the non-specific PDE inhibitor IBMX (1 mM) and the allosteric enhancer of NO receptor-guanylyl cyclase BAY 41-2272 (3 µM) were immunolabelled for cGMP (green) and counterstained using DAPI (blue nuclei). **A.** Composite image of an entire hippocampal section. **B.** Double labelling for the astrocyte marker GFAP (red, left) and cGMP (green, middle) in the CA1 subfield. Colocalisation appears in yellow in the right-hand image. Arrows without tails indicate double-labelled cells and arrows with round tails, cGMP-positive, GFAP-negative cells. **C, D.** Slices were pre-treated with the PDE-2 inhibitor BAY 60-7550 (10 nM) instead of IBMX. Photographs are as in A and B. The number of GFAP-positive fibres in D appears to be fewer than in B, because of the oblique plane of section through the cell layer. Key: bv, blood vessel; DG, dentate gyrus; gr, granule cell layer; h, hilus; lu, stratum lucidum; mol, stratum moleculare; or, stratum oriens; pyr, stratum pyramidale; rad, stratum radiatum; Sub, subiculum. Scale bar in A = 200 µm (also applies to panel C); B = 30 µm; D = 50 µm.

Initial experiments aimed to reproduce conditions previously used with adult hippocampal slices to determine if the targets of NO might be age-dependent. The slices were pre-incubated with the general PDE inhibitor IBMX (1 mM) and the allosteric enhancer of NO-targeted guanylyl cyclase BAY 41-2272 (3 µM; [31]). Slices were then fixed, sectioned and immunolabelled for cGMP. In good agreement with the results of previous histochemical studies on adult tissue [19]–[26], cGMP immunofluorescence was detected in some blood vessels and a population of scattered cells and fibres that were predominant in the stratum radiatum and oriens of the CA1 subfield, most cells in the stratum pyramidale being immunonegative (Figure 1A). Using double immunofluorescent staining, many immunopositive cells were found to express the astrocyte marker, glial fibrillary acidic protein (GFAP; Figure 1B), consistent with previous reports on adult rodent hippocampus [25], [26]. GFAP-negative, cGMP-positive cells, which may be interneurones or other types of glia, were also observed. In accordance with PDE-2 being primarily responsible for the breakdown of cGMP in the hippocampus [26], [27], a similar intensity and distribution of cGMP immunofluorescence was observed using the PDE-2 inhibitor BAY 60-7550 (10 nM; [19]) in place of IBMX (Figure 1C–D).

**Figure 2:**
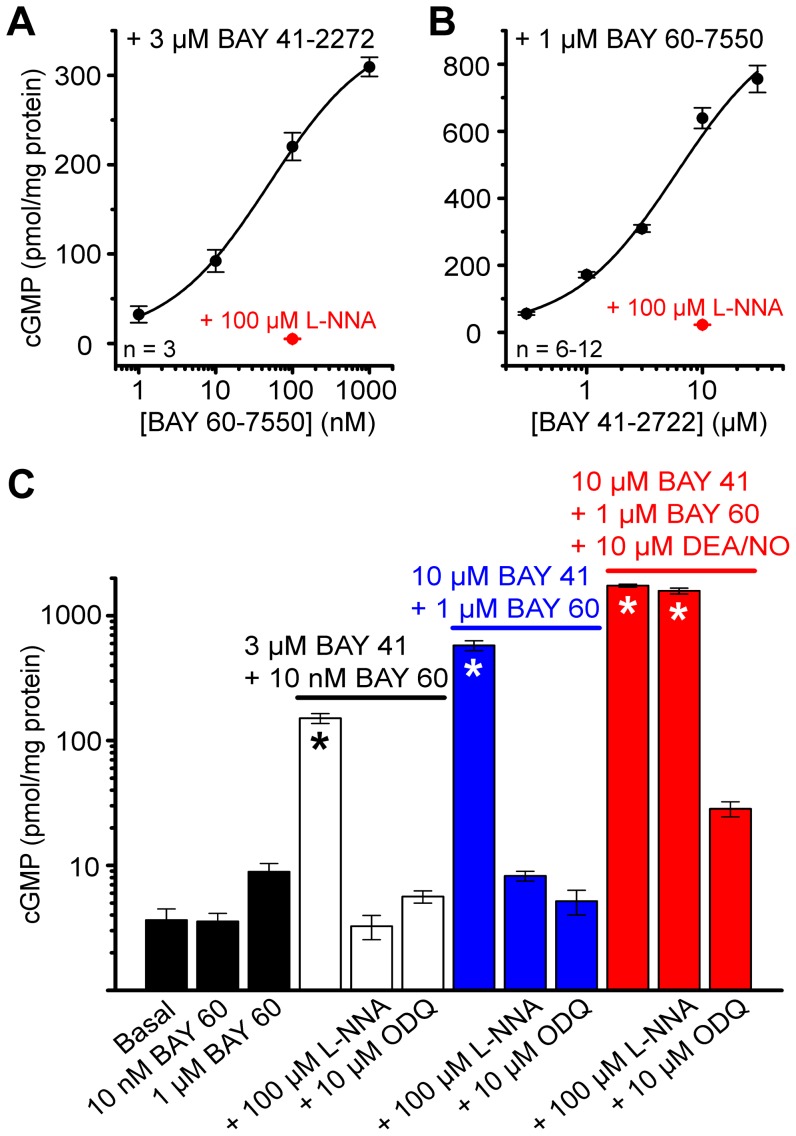
Quantification of cGMP accumulation. **A.** Concentration-cGMP response curve for BAY 60-7550 in the presence of BAY 41-2272 in immature rat hippocampal slices. The EC_50_ value was 50±9 nM and the Hill Slope was 0.65±0.04. **B.** Concentration-cGMP response curve for BAY 41-2722 in the presence of BAY 60-7550. EC_50_ and Hill slope values were 6±3 µM and 0.96±0.12. In A and B, data shown in red were collected from slices that were additionally pre-treated with the NOS inhibitor L-NNA. Fits are logistic; adjusted R^2^ statistics of the fits were >0.98. **C.** Effect of BAY compounds and the NO donor DEA/NO on cGMP. Data shown in white, blue and red were collected in parallel with the data shown in Figure 1C, 3A and 4A. Experimental conditions are indicated by the colour-coded labels. In every experiment, basal cGMP levels and the effect of L-NNA or the NO receptor antagonist ODQ on the cGMP response were measured. Basal levels were pooled across experiments. Asterisks: *p*<0.05 compared to basal by ANOVA with Dunnett's *post hoc* test. *n* = 4–12.

To calibrate the conditions tested above, a concentration-cGMP response curve for BAY 60-7550 (in the presence of 3 µM BAY 41-2722) was compiled. The results showed that the concentration of BAY 60-7550 used in the above experiments (10 nM) was far from optimal, the EC_50_ value being around 50 nM (Figure 2A). A similar concentration-response curve for the allosteric enhancer BAY 41-2722 in the presence of a near-maximal concentration of BAY 60-7550 (1 µM), gave an EC_50_ of about 6 µM (Figure 2B). With near-maximal concentrations of both compounds (10 µM BAY 41-2272 and 1 µM BAY 60-7550) the amount of cGMP generated was 4-fold higher than in slices pretreated with the previously-used concentrations of both BAY compounds (Figure 2A–C, compare white and blue bars).

**Figure 3:**
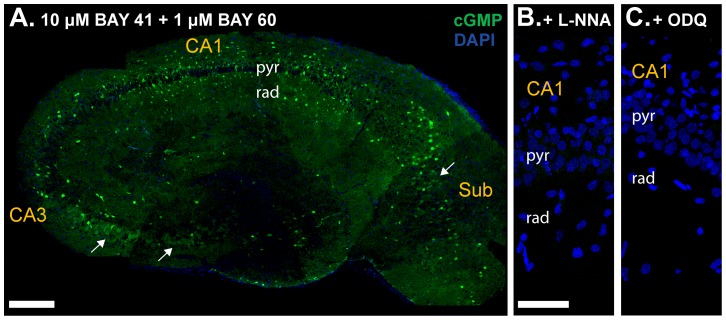
cGMP immunofluorescence with near-maximal concentrations of both BAY compounds. Sections of slices pre-incubated with BAY 60-7750 (1 µM) and BAY 41-2272 (10 µM) were immunolabelled for cGMP (green) and counterstained with DAPI (blue). **A.** Composite image of an entire hippocampal section. Arrows mark pyramidal cell staining. **B, C.** cGMP immunolabelling was prevented by the NOS inhibitor L-NNA (100 µM; **B**) or the NO receptor antagonist ODQ (10 µM; **C**). Photographs show area CA1. For key, see Figure 1 legend. Scale bar in A = 200 µm; B = 50 µm (applies also to C).

When cGMP immunohistochemistry was repeated using the improved conditions, there was a striking increase in the area and intensity of cGMP staining (Figure 3A). In addition to pronounced staining now appearing in the neuropil of all subfields, scattered cells and blood vessels, a population of pyramidal neurones (identified by their number, position and morphology) in the subiculum and, more obviously, in area CA3/CA4 were immunolabelled. Labelling throughout the dentate gyrus remained comparatively weak. The cGMP staining was entirely prevented by inhibition of NOS (L-NNA, 100 µM; (Figure 3B), showing that the response was dependent on endogenously produced NO and consistent with BAY 41-2722 acting as an allosteric enhancer to potentiate the action of NO on its receptors [32]. The immunostaining was also abolished by ODQ (10 µM), an NO receptor antagonist (Figure 3C). L-NNA and ODQ similarly blocked the cGMP response of whole hippocampal slices, as measured by radioimmunoassay (Figure 2C, blue bars).

**Figure 4:**
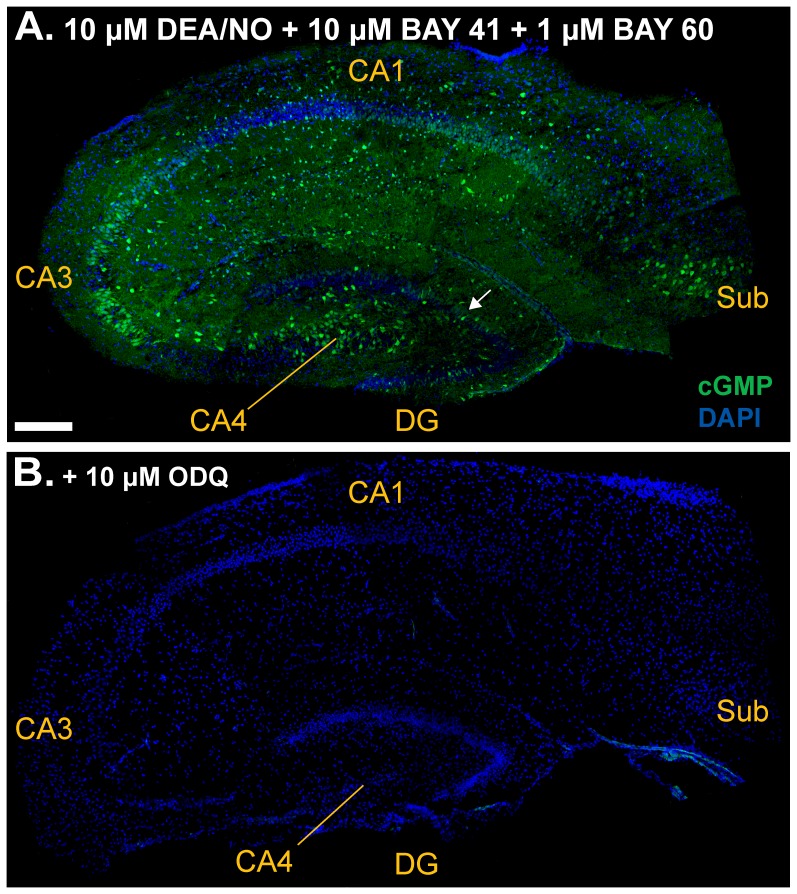
Effect of supplementary NO on cGMP immunofluorescence. Slices were exposed to the NO donor DEA/NO (10 µM) in the presence of 1 µM BAY 60-7550 and 10 µM BAY 41-2272. Sections were immunolabelled for cGMP (green) and counterstained with DAPI (blue). **A.** Composite image showing an entire hippocampal section. White arrow: example of cGMP staining in a patch of granule cells. **B.** Composite image of a whole section of a slice that was also pretreated with ODQ (10 µM). For key, see Figure 1 legend. Scale bar in A = 200 µm for both panels.

Given that the conditions so far examined are dependent on basal NO production within the tissue, and that basal NO concentrations are low relative to those seen on stimulation of nNOS, for example with NMDA [33], the effect of adding supplementary NO was tested. To do so, a concentration (10 µM) of the NO donor DEA/NO that is maximal for cGMP accumulation in adult rat hippocampal slices [9] was co-applied with BAY 60-7550 (1 µM) and BAY 41-2272 (10 µM). Under these conditions, cGMP levels measured using radioimmunoassay were further increased by a factor of 3 (Figure 2C, red bars). Consistent with this enhancement in overall slice cGMP, immunohistochemistry showed that the intensity, as well as the area, of cGMP immunofluorescent staining was augmented (Figure 4A). Most obviously, the majority of pyramidal cells in CA3 and a distinct proportion of those in CA1were now stained, as were numerous neurone-like cells in CA4 and the subiculum. Some granule cells in the dentate gyrus also showed weak staining. Again, the cGMP elevations detected both by radioimmunoassay and by immunohistochemistry were prevented by ODQ (10 µM; Figure 2C and 4B).

**Figure 5:**
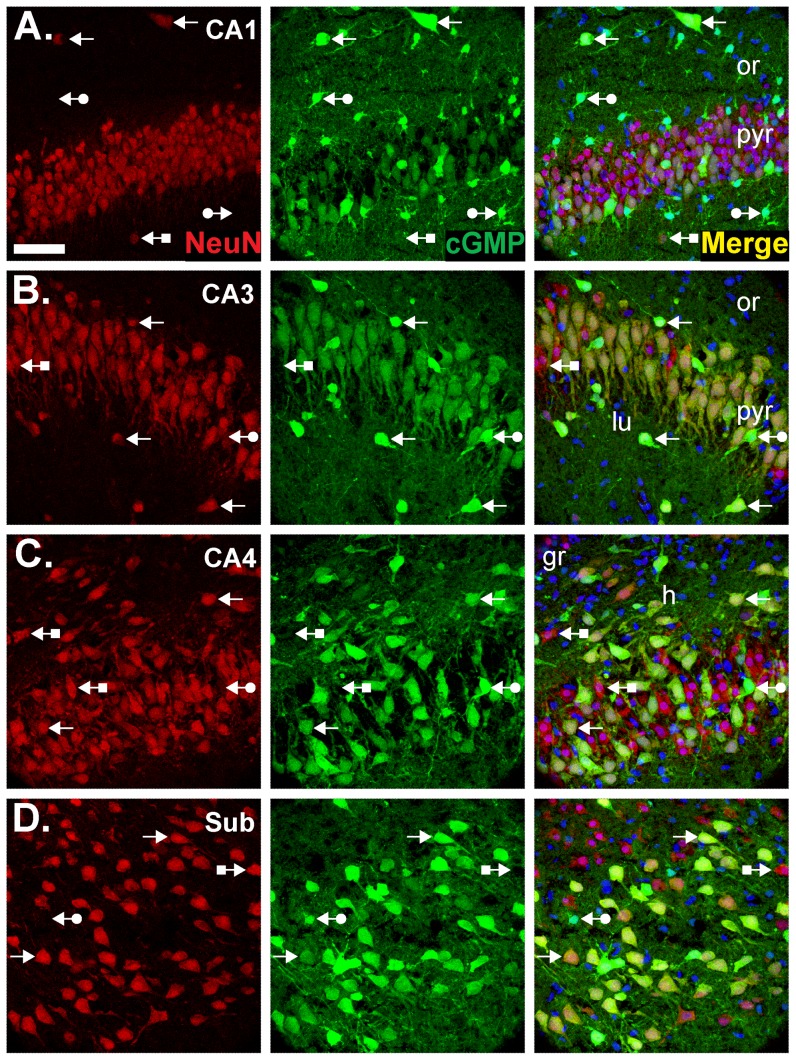
Double labelling for cGMP and the neuronal marker, NeuN. Experimental conditions were as in Figure 4A (1 µM BAY 60-7550, 10 µM BAY 41-2272, 10 µM DEA/NO). Sections were labelled for NeuN (red, left) and cGMP (green, middle) and counterstained with DAPI (blue). Images show CA1 (**A**), CA3 (**B**), CA4 (**C**) and the subiculum (**D**). Colocalisation appears yellow in the right-hand images. Arrows without tails indicate double-labelled cells; arrows with round tails, cGMP-positive, NeuN-negative cells; arrows with square tails, cGMP-negative, NeuN-positive cells. See Figure 1 legend for the key. The scale bar in A = 50 µm and applies to all panels.

To provide a more detailed anatomical picture, experiments in which slices were treated with DEA/NO (as above) were repeated and sections from them subjected to dual labelling for cGMP and some standard cellular markers. NeuN was used to identify neurones, preferentially staining their nuclei. Pyramidal neurones throughout the hippocampus were co-stained, the intensity and frequency of staining being in the order CA3 and subiculum>CA4>CA1 (Figure 5). Many NeuN-positive cells lying outside the pyramidal cell layer displayed very bright cGMP labelling, indicating cGMP synthesis in interneurones. Nevertheless, some cells staining for NeuN were immunonegative for cGMP, and vice versa.

**Figure 6:**
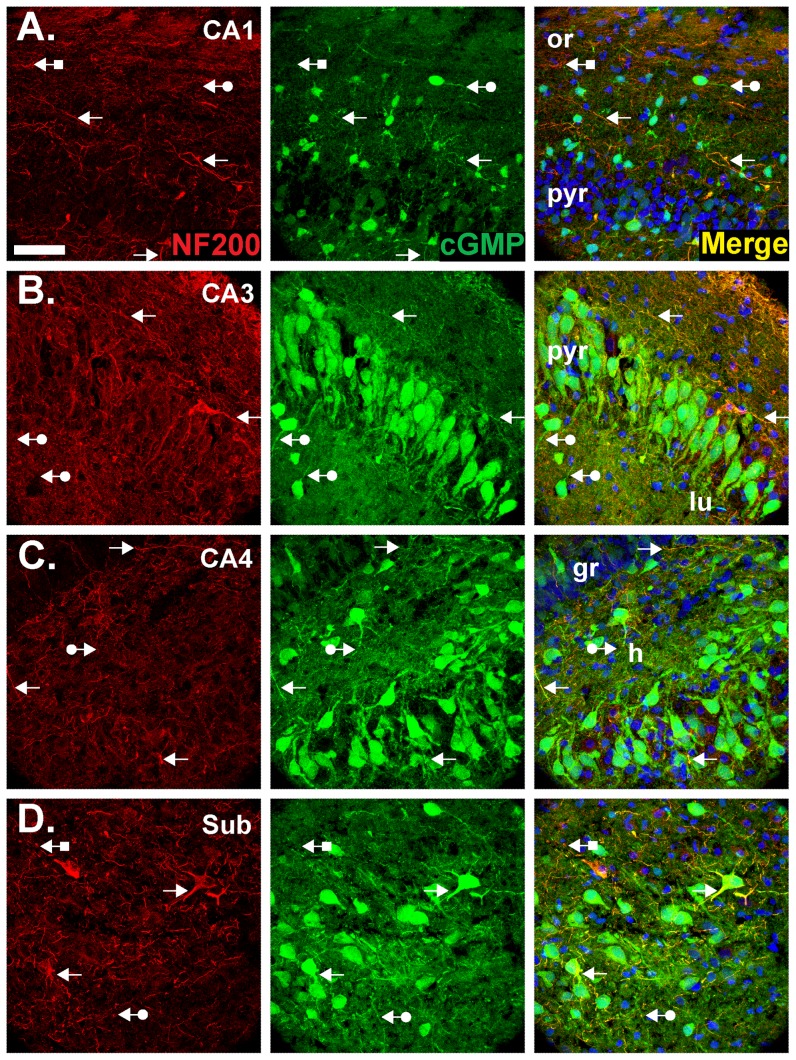
Double labelling for cGMP and the axonal marker, NF200. Experimental conditions were as in Figure 4A and 5 (1 µM BAY 60-7550, 10 µM BAY 41-2272, 10 µM DEA/NO). Sections were labelled for NF200 (red, left) and cGMP (green, middle) and were counterstained with DAPI (blue). Images show CA1 (**A**), CA3 (**B**), CA4 (**C**) and the subiculum (**D**). Colocalisation appears yellow in the right-hand images. Arrows without tails indicate double-labelled fibres; arrows with round tails, cGMP-positive, NF200-negative fibres; arrows with square tails, cGMP-negative, NF200-positive fibres. See Figure 1 legend for the key. The scale bar in A = 50 µm and applies to all panels.

Fibres expressing the marker for axons, neurofilament-200 (NF200), were also found to accumulate cGMP. Co-localisation was most obvious in CA1 (Figure 6A), CA3 (Figure 6B) and the subiculum (Figure 6D) in the strata oriens, pyramidale and radiatum. In CA4, cGMP-immunopositive axons were evident in the hilus (Figure 6C). Throughout the tissue, cGMP-immunopositive, NF200-negative fibres were also visible, presumably reflecting cGMP accumulation in astrocyte processes or dendrites, as were NF200-positive, cGMP-negative structures.

**Figure 7:**
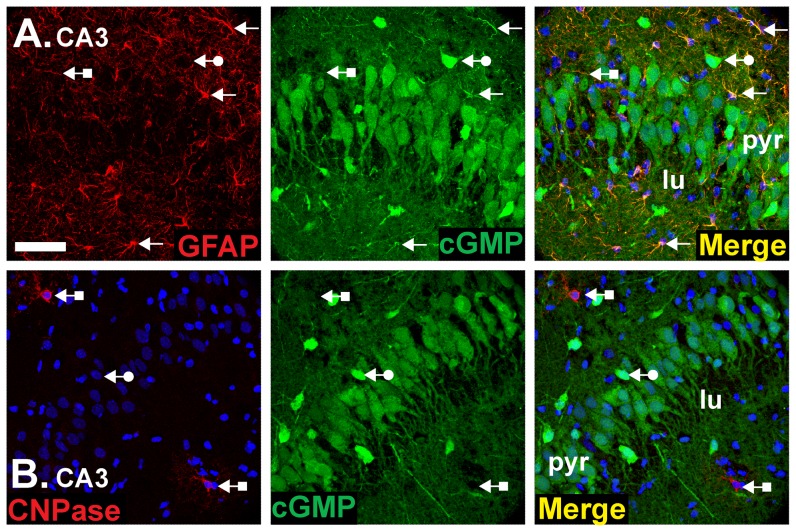
Double labelling for cGMP and glial cell markers. Experimental conditions were as in Figure 4A, 5 and 6 (1 µM BAY 60-7550, 10 µM BAY 41-2272, 10 µM DEA/NO). Sections were doubled-labelled for cGMP (green) and a marker for astrocytes (GFAP; red in **A**) or oligodendrocytes (CNPase; red in **B**). Photographs show an area of the CA3 subfield and are representative of findings throughout the hippocampus. Arrows without tails: double-labelled cells and fibres; arrows with round tails, cGMP-positive, glial cell marker-negative cells; arrows with square tails, cGMP-negative, glial cell marker-positive cells. The key is as in Figure 1 legend. Scale bar in A = 50 µm and applies to both panels.

As expected from previous findings (Figure 1B and D; [25], [26]), cGMP was also found associated with astrocytes, as indicated by co-staining for GFAP (Figure 7A). Oligodendrocytes expressing the myelin-associated marker 2′,3′-cyclic-nucleotide 3′-PDE (CNPase), by contrast, were cGMP immunonegative (Figure 7B).

### Immunohistochemistry for NO-targeted guanylyl cyclase subunits

**Figure 8:**
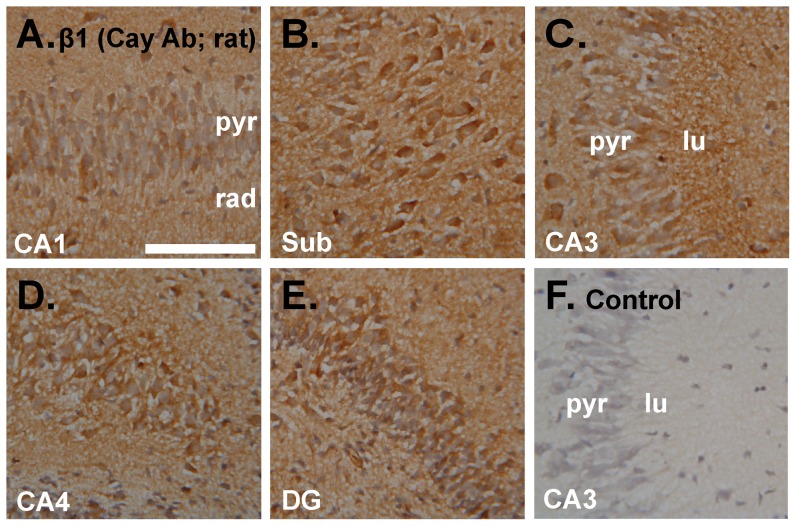
Immunoperoxidase staining using an antibody raised against the NO receptor-guanylyl cyclase β1 subunit. **A–E.** Incubated immature rat hippocampal slices were sectioned and stained for the β1 subunit (brown) using a primary antibody from Cayman Chemical Company (Cay Ab). Photographs show an area of CA1 (**A**), the subiculum (sub; **B**), CA3 (**C**), CA4 (**D**) and the dentate gyrus (**E**). **F.** Control with the primary antibody omitted showing area CA3 (representative of the entire hippocampus). Tissues were fixed in 1% paraformaldehyde. See Figure 1 legend for key. Scale bar in F = 100 µm and applies to A–F.

In an attempt to determine if the results are compatible with the distribution of NO receptor protein, immunoperoxidase staining for the common β1 guanylyl cyclase subunit was carried out in the same tissue (i.e. incubated immature rat hippocampal slices). As indicated using an antibody from Cayman Chemical Company (Table S1), the β1 subunit was present in all regions, including in pyramidal and dentate granule cells, and in the neuropil generally (Figure 8).

The common β1 subunit exists in combination with either an α1 or α2 subunit to generate an active receptor-enzyme [5]. Both of these α-subunits are required for LTP at hippocampal CA1 synapses but may perform different roles [10] and so knowledge of their location relative to the sites generating cGMP is desirable. Unfortunately, an antibody for the α2-subunit is not available but one for the α1-subunit (Table S1) has been widely used for immunohistochemistry, including in the hippocampus [17], [28], [34]–[38]. Having access to brain tissue from mice lacking the α1-subunit afforded us the opportunity to test the specificity of this antibody.

**Figure 9:**
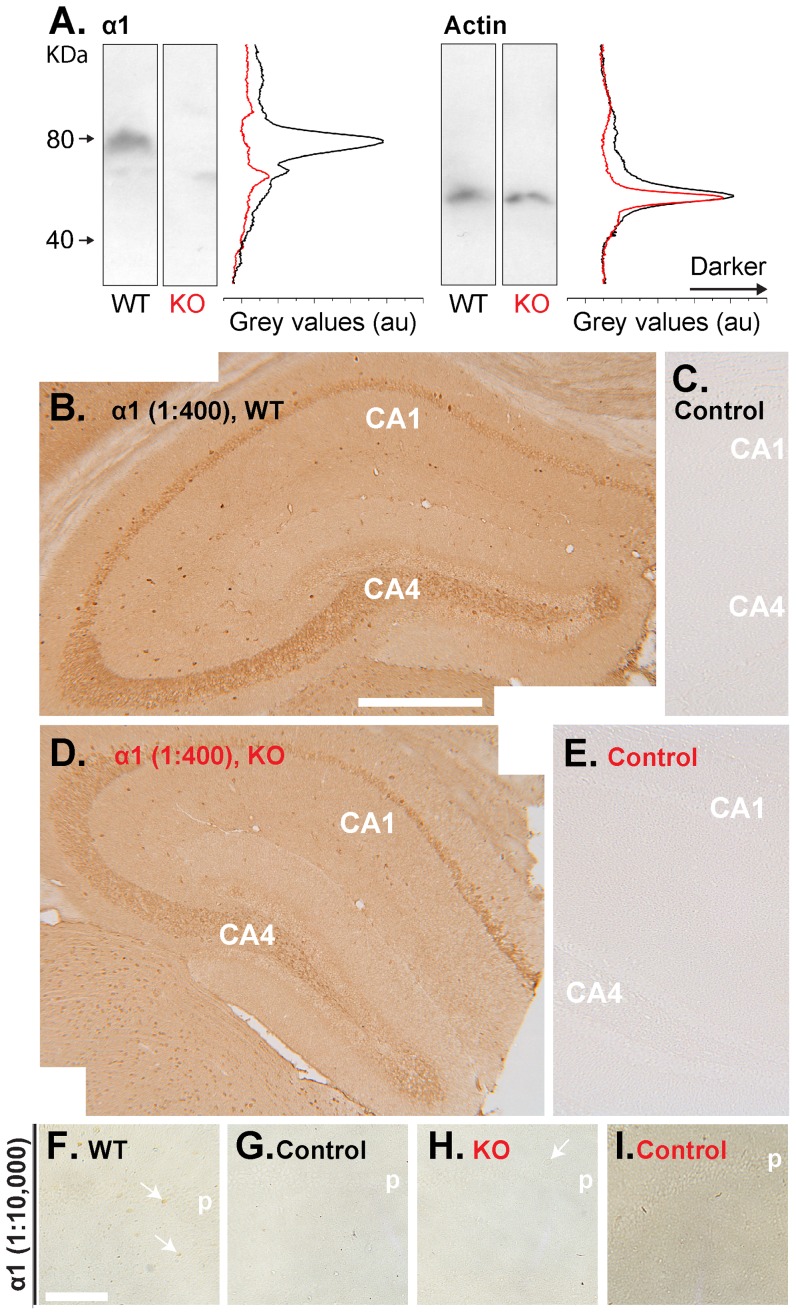
Evaluation of an antibody raised against the NO receptor-guanylyl cyclase α1 subunit. Experiments were done using adult mice. **A.** Western blots of lysates of wild-type (WT, black) and α1-knockout (KO, black) cerebellum were probed for the α1 protein (left) or, after removing the α1 antibody, actin (right). Grey values (given in arbitrary units; au) for each horizontal row of pixels in the wild-type and knockout lanes are shown in black (WT) and red (KO). Molecular weights (kDa, far left) apply to both panels. Lysates from wild-type and knockout animals were processed in parallel on the same gels/membranes. **B–E.** Hippocampal sections prepared from the wild-type (WT; **B**) and α1-knockout (KO; **D**) mice used in A were stained (brown) using an antibody for the α1 subunit diluted 1∶400. Images are a composite of two photographs. Controls (same orientation as experimental sections) with the primary antibody omitted are in C and E. **F–I.** Staining of sections of wild-type (**F**) and α1-knockout (**H**) hippocampus (CA1 area) using a 1∶10,000 dilution of the α1 antibody. **G** and **I** are controls (primary antibody omitted). Knockout and wild-type tissues were processed in parallel and, for clarity, tissues were not counterstained. The experimenter was blinded to genotype until all the photographs were taken. Key: p = stratum pyramidale. Scale bar in B = 500 µm and applies to B–E; scale in F = 155 µm is for F–I.

Encouragingly, in western blots of wild-type tissue, the α1-antibody gave a single band of the correct molecular weight (approximately 80 kDa) that was absent in the knockout tissue (Figure 9A). When used in immunohistochemistry at a 1∶400 dilution, the α1 antibody gave staining in pyramidal neurones, blood vessels, neuropil and cells scattered throughout sections of wild-type adult mouse hippocampus (Figure 9B). However, the staining was identical in sections from α1-knockout mice (Figure 9D). The lack of specific staining was unrelated to the dilution of the antibody, as a 1∶10,000 dilution, which was just threshold for visible staining (primarily in a few scattered cells), generated similar staining in the wild-type and knockout tissues (Figure 9F–I). Further tests using the antibody with sections from cerebellum and cerebral cortex showed similar non-specific staining, with the exception of the cerebellar pia mater where the staining was missing in the knockout animals (Figure S1). The specificity seen in western blots, contrasting with non-specificity in immunohistochemistry, is reminiscent of findings using an antibody against an NMDA receptor subunit in the hippocampus [39]. Moreover, the non-specific staining pattern reported in this previous study (their Figure 3) is very similar to that seen here (Figure 9B and D). An epitope unmasking technique (pretreating the sections with pepsin) found to expose specific staining with the NMDA receptor antibody was, however, unsuccessful when used with the α1 antibody (Figure S2).

These findings raise questions over the specificity of the β1 antibody (Figure 8), which gave a similar pattern of staining to the α1. β1-knockout mice were not available to us and are difficult to obtain because of the high incidence of foetal and postnatal fatality [40]. As an alternative, a second β1 antibody (Table S1; [25]) was used. When tested in parallel in adult mouse hippocampus, the two β1 antibodies gave comparable staining patterns (Figure S3). An exception was the stratum lucidum in the CA3 subfield, which was stained more heavily with one than the other.

## Discussion

The principal findings of the present study are that, as judged by cGMP immunohistochemistry, the targets of NO in the hippocampus are much more widespread than previous evidence of this type had suggested and, in particular, that the principal neurones participating directly in NO-dependent LTP are NO-responders, rendering unnecessary more complicated hypotheses invoking the participation of other cells. Further, results aimed at determining the immunolocation of NO receptor protein raise doubts about the veracity of current antibodies used for this purpose, although our data obtained using cGMP immunohistochemistry are generally in accordance with predictions based on the distribution of NO receptor subunit mRNA, as studied by *in situ* hybridization.

No previous investigation of the location of cGMP by immunohistochemistry in hippocampal slices has succeeded in detecting the nucleotide in pyramidal neurones after exposure to NO in combination with various PDE inhibitors [17]–[26]. We could broadly replicate the results of these past studies (Figure 1) but it transpired that the conditions used were substantially submaximal for cGMP accumulation (Figure 2), suggesting that the differences in pyramidal cell staining are simply explicable on the basis of the size of the cGMP signal relative to the detection threshold of the immunohistochemical method.

Previously, the highest level of cGMP accumulation reported in hippocampal slices in response to NO in the presence of PDE inhibitors (typically IBMX) was around 60 pmol/mg protein [19] with values in the range 20–50 pmol/mg protein being common in studies where cGMP immunohistochemistry was conducted in parallel [24], [26]. Assuming a protein concentration of 100 mg/ml in brain (taking total protein to be 10% of weight and a tissue density of 1 g/ml), 20–30 pmol/mg protein translates into an overall cGMP concentration of 2–3 µM. Given that the cGMP antibody used for immunofluorescence has a detection limit of about 10 µM [41], [42], it is unsurprising that the extent of visible immunohistochemical labelling was limited. The predominance of staining in astrocytes observed in previous studies [25], [26] and in Figure 1 may reflect a low PDE/high guanylyl cyclase activity of these cells, reminiscent of astrocytes in the cerebellum where, even in the absence of a PDE inhibitor, cGMP can reach near-millimolar concentrations [43]. From measurements in the hippocampal stratum radiatum [44], astrocytes take up only 4–8% of the volume, so these cells having cGMP concentrations above the threshold for detection would still be compatible with an overall cGMP concentration of 2–3 µM.

In marked contrast to these previous findings, the cGMP level we observed under optimal conditions was around 2 orders of magnitude higher, at around 1700 pmol/mg protein (Figure 2). This value appears to be the highest recorded for brain, surpassing even maximal NO-evoked cGMP responses in the cerebellum, which are around 1000 pmol/mg protein [45] and comparable to the response amplitude observed in rat platelets, a pure population of NO-responder cells, in the presence of a PDE inhibitor [46], [47]. With an overall corresponding tissue cGMP concentration of around 170 µM, it is predictable that the immunofluorescent signal would be more intense and widespread (Figure 4).

Two methodological factors are likely to have favoured the accumulation of higher levels of cGMP in the present study. First, we used immature rat hippocampal slices, which have been shown to generate around 5-fold more NO-dependent cGMP in response to NMDA than those of the adult [30]. With few exceptions [17], [18], previous tests of the location of NO-evoked cGMP in the hippocampus have been made using slices prepared from adult rats [19]–[22], [24], [26] and/or mice [23], [25]. The second factor is the particular combination of compounds used to promote cGMP accumulation: BAY 60-7550 to inhibit PDE-2, the major cGMP-degrading enzyme in hippocampus [26], [27], BAY 41-2772 to increase the maximal NO-evoked guanylyl cyclase activity [31], [32], and DEA/NO to deliver authentic NO. This combination had not been tested previously.

Even without supplementary NO, hippocampal cGMP was comparatively high (580 pmol/mg protein; Figure 2), reflecting endogenous NO acting under the potentiating influence of BAY 41-2272. From experiments on purified NO-activated guanylyl cyclase [32], 10 µM BAY 41-2272 shifts the NO concentration-response curve to the left by a factor of at least 20, enabling marked stimulation of guanylyl cyclase by the picomolar NO concentrations purported to exist in unstimulated brain slices [9]. Much of this resting NO concentration appears to be derived from eNOS, which is capable of generating NO tonically over periods of hours as a result of phosphorylation of the enzyme by the kinase Akt [9], [48]. Immunohistochemically, the difference in cGMP labelling in response to endogenous and exogenous NO was mainly one of degree (i.e. staining more intense and/or encompassing more of the pyramidal cell layer; compare Figure 3 and 4), suggesting that exogenous NO exposes targets that would be accessed by higher levels of endogenously-generated NO, which potentially rise to the low nanomolar range on nNOS stimulation [33]. It is unclear why pyramidal cell somata (and granule cell somata in the dentate gyrus) were the most resistant to the accumulation of cGMP into the detectable range. High PDE activity and/or low NO-activated guanylyl cyclase activity and/or the signalling pathway being concentrated at sites distant from the cell bodies are possible reasons.

The widespread distribution of cGMP immunoreactivity in the hippocampus in response to NO implies that most of the constituent cellular elements are potential NO targets, including pyramidal neurones, interneurones, astrocytes and at least some dentate granule cells. Some cells, notably CNPase-positive oligodendrocytes (Figure 7B) and some presumed interneurones (Figure 5), remained cGMP-immunonegative but their failure to accumulate cGMP to detectable levels does not necessarily exclude them as targets of NO, although some interneurones genuinely appear to lack NO receptor subunits [35]: for example, their dominant PDE may not be PDE-2.

Other approaches to the identification of NO targets in the hippocampus are *in situ* hybridization and immunocytochemistry for the NO receptive guanylyl cyclase subunits. In the immature rat hippocampus [49], like in the adult [50], mRNA for the NO receptor is widespread. The common β1 subunit and the α2 subunit appear to be strongly expressed in the pyramidal cell layer and in the granule cells of the dentate gyrus [49]. The results of Pifarre *et al.* [51] also indicate expression of α2 subunit mRNA in scattered cells, presumably interneurones and/or glial cells (their Figure 4B) whereas others report that this subunit is restricted to pyramidal neurones [35]. The α1-subunit mRNA shows a more diffuse distribution, reportedly being exclusively expressed in subpopulations of interneurones in one study [35] but also detected in pyramidal cells in another [51]. Irrespective of these discrepancies in detail, our results are consistent with the broad expression of NO-activated guanylyl cyclase indicated by *in situ* hybridization.

There have been several descriptions of the distribution of NO-activated guanylyl cyclase subunits in the hippocampus using immunohistochemistry. Our results with two different antibodies against the common β1-subunit are in general agreement with each other (Figure 8 and S3) and with previous findings using this method [25], [52], [53]. The β1-protein distribution is also compatible with the mRNA distribution (see above) and with our cGMP immunofluorescence results (Figure 4), signifying that the protein detected by immunohistochemistry is genuine. However, this conclusion is weakened by the fact that a similar distribution obtained using an antibody against the α1-subunit proved non-specific (Figure 9B–E). This antibody has been the only one used to map the location of α1-protein beforehand and has led to the conclusion that the α1β1 isoform is only in interneurones [17], [35]. Whilst consistent with the *in situ* hybridization result carried out by the same group [35], and with our results with the same antibody used at a similar dilution (1∶10,000), the persistence of the staining in α1-knockout tissue raises concerns about the authenticity of the staining (Figure 9F–I). Nevertheless, populations of interneurones did display particularly intense cGMP immunoreactivity in our study (Figure 5).

In the hippocampus, NO signalling has been most studied in the context of LTP in the CA1 subfield and current evidence suggests that after its formation by nNOS postsynaptically, NO acts both pre- and postsynaptically to bring about a coordinated and persistent increase in synaptic efficacy. Postsynaptically, functional evidence indicates that NO, through cGMP, triggers gene expression [14] and the insertion of AMPA receptors into the cell membrane [15]. By showing cGMP accumulation in at least some CA1 pyramidal neurones (Figure 4), our results provide anatomical support for a postsynaptic site of action. Higher resolution methods will be needed to explore the subcellular locations of cGMP but there was obvious staining of the apical dendrites of CA3 neurones (Figure 5B, 6B and 7) and, to judge from the lack of empty dendritic profiles extending into stratum radiatum in the CA1 subfield (Figure 4, 5A, 6A), it is probable that pyramidal cell dendrites are NO-responsive. Immunohistochemistry for the β1-subunit of NO-activated guanylyl cyclase also produces staining in CA1 dendrites ([25]; Figure S3). Presynaptically, NO reportedly increases neurotransmitter release through cGMP [11], [12]. Consistent with this site of action, cGMP labelling was seen in axons throughout the hippocampus (Figure 6) but, again, higher resolution would be needed to determine if cGMP accumulates in nerve terminals specifically. Also consistent with a presynaptic effect of NO, examination of the location of the β1 NO receptor subunit by immunohistochemistry reports presynaptic staining in some synapses [52], [54], as do studies showing NO-evoked cGMP accumulation in some nerve terminals [19], [25]. Hence, an anatomical picture that coheres with functional evidence with respect to hippocampal NO-cGMP signalling and synaptic plasticity is beginning to taking shape.

## Supporting Information

Table S1 **Primary antibodies used.** ^a^ University of Maastricht, The Netherlands; ^b^ for immunoperoxidase staining; ^c^ for western blotting; ^d^ for adult mouse hippocampus; ^e^ for immature rat hippocampus; ^f^ Technische Universitat Braunschweig, Germany.
(DOC)

Figure S1 **Immunoperoxidase staining using the α1 antibody in sections of cerebellum and cerebral cortex.** Sagittal cerebellar and transverse cerebral cortex sections were prepared from wild-type (WT) and α1 knockout (KO) mice and immunostained (brown) by the same methods used for mouse hippocampal sections. The α1 antibody was diluted 1∶400. **A–D.** Photographs show sections of WT (**A**) and KO (**C**) cerebellum. **B** and **D** are controls (primary antibody omitted). Arrows point to the pia mater. For clarity, tissues were not counterstained. **E–H.** WT (**E**) and KO (**F–H**) cerebral cortex. Insets: controls (no primary antibody) showing approximately the same regions as in the larger photographs. Tissues were counterstained with Mayer's hemalum (blue). Key: gr, stratum granulare; mol, stratum moleculare; Purkinje, Purkinje cell layer; wm, white matter. Scale bar in A = 200 µm and applies to A–D; scale in E = 100 µm and applies to E–H.
(TIF)

Figure S2 **Effect of pepsin pretreatment on the specificity of immunostaining using the α1 antibody.** Transverse hippocampal sections prepared from wild-type and α1 knockout mice were rehydrated using TBS (5 min) and then treated with 2 mg/ml pepsin (prepared in 30 mM HCl) for 10 min at 37°C according to published methods for epitope unmasking [38]. Tissues were then stained using the immunoperoxidase method. The α1 antibody was used at a dilution that was just suprathreshold for visible staining (1∶10,000). **A–D**. Area CA1 of wild-type (**A**) and knockout (**C**) tissue. The primary antibody was omitted in **B** and **D**. Sections were not counterstained. Key: pyr, stratum pyramidale; rad, stratum radiatum. For all panels, the scale bar = 200 µm. In other experiments, the pepsin concentration was altered to 0.2 or 4 mg/ml. Under these conditions, the distribution of staining varied from that shown but was similarly non-specific.
(TIF)

Figure S3 **Immunoperoxidase staining in adult mouse hippocampus using different antibodies for the β1 guanylyl cyclase subunit.** **A.** Staining for β1 (brown) using the Cayman antibody in a section fixed with 1% paraformaldehyde. Image is a composite of two photographs. **B.** Control for A (no primary antibody); arrows point to non-specific staining. **C.** Composite image of β1 staining using an antibody provided by S. Behrends. **D.** Control for C (no primary antibody). Tissues were counterstained with Mayer's hemalum (blue). Scale bar in A = 500 µm and applies to all panels.
(TIF)
