# Supplementary material for: Cellular Targets of Nitric Oxide in the Hippocampus
Source: PLoS One. 2013 Feb 25;8(2):e57292. doi: 10.1371/journal.pone.0057292 (PMC3581475; doi:10.1371/journal.pone.0057292)
Supplement: Table S1 — Primary antibodies used. a University of Maastricht, The Netherlands; b for immunoperoxidase staining; c for western blotting; d for adult mouse hippocampus; e for immature rat hippocampus; f Technische Universitat Braunschweig, Germany. (DOC) [file pone.0057292.s001.doc]

**Table S1. Primary antibodies used.**

| **Antigen** | **Host** | **Final dilution** | **Product code and/or supplier** |
| --- | --- | --- | --- |
| Actin 1-19 | Goat | 1:500 | SC-1616, Santa Cruz Biotechnology |
| cGMP | Sheep | 1: 40,000 | J. de Vente^a^ |
| CNPase | Mouse | 1:2,000 | MAB 326, Chemicon Europe |
| GFAP | Rabbit | 1:1,000 | MAB 360, Chemicon Europe |
| NeuN | Mouse | 1:1,200 | MAB 377, Chemicon Europe |
| NF200 | Mouse | 1:500 | MAB 1623, Chemicon Europe |
| α1 | Rabbit | 1:400-1:10,000^b^, 1:500^c^ | G4280, Sigma-Aldrich Company |
| β1 | Rabbit | 1:250^d^, 1:500^e^ | CAY-160897-1, Cayman Chemical |
| β1 | Rabbit | 1:600 | S. Behrends^f^ |

^a^ University of Maastricht, The Netherlands; ^b^ for immunoperoxidase staining; ^c^ for western blotting; ^d^ for adult mouse hippocampus; ^e^ for immature rat hippocampus; ^f^ Technische Universitat Braunschweig, Germany.
