# Supplementary figures and images for: Cellular Targets of Nitric Oxide in the Hippocampus
Source: PLoS One. 2013 Feb 25;8(2):e57292. doi: 10.1371/journal.pone.0057292 (PMC3581475; doi:10.1371/journal.pone.0057292)

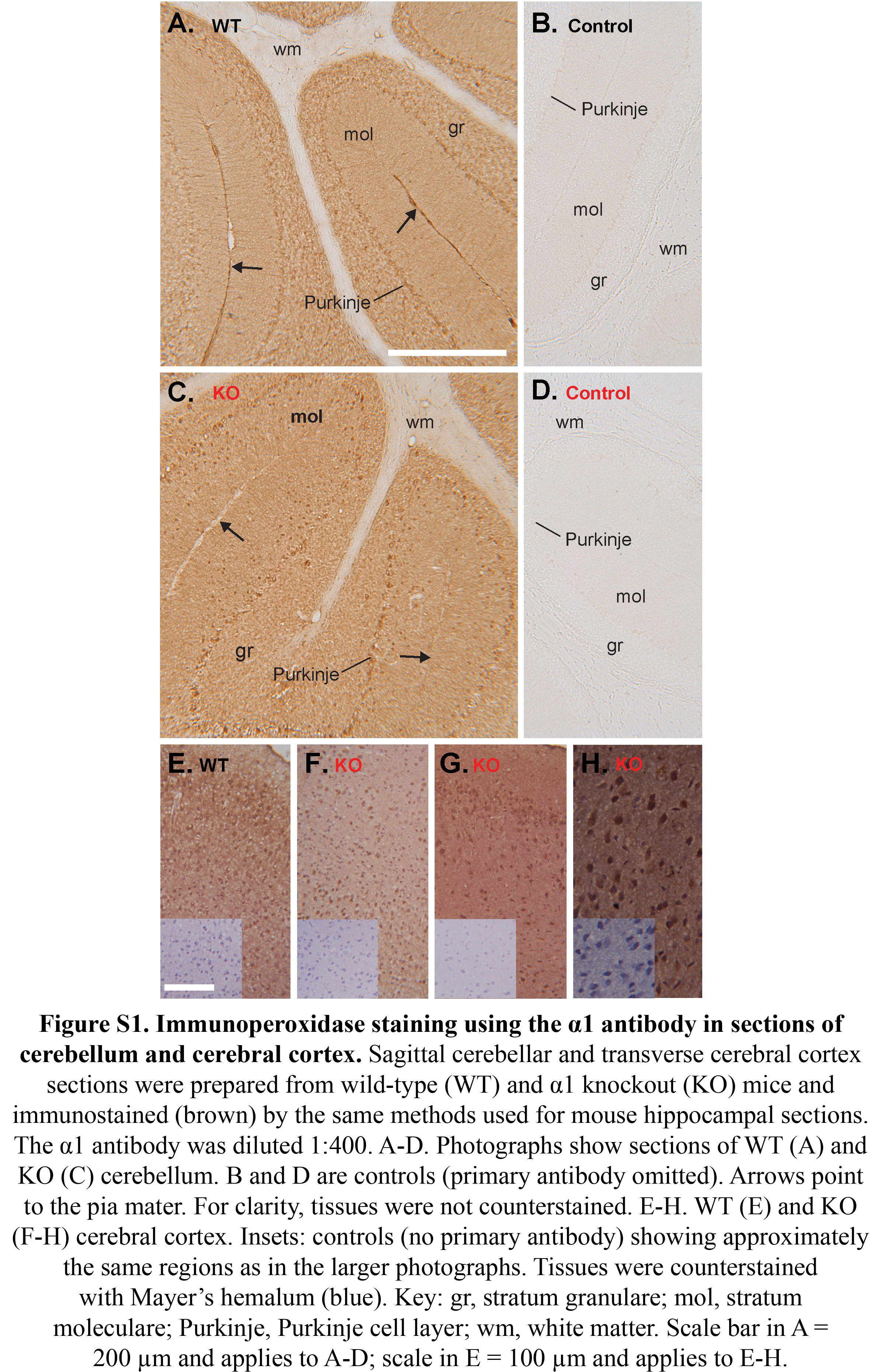

Supplement: Figure S1 — Immunoperoxidase staining using the α1 antibody in sections of cerebellum and cerebral cortex. Sagittal cerebellar and transverse cerebral cortex sections were prepared from wild-type (WT) and α1 knockout (KO) mice and immunostained (brown) by the same methods used for mouse hippocampal sections. The α1 antibody was diluted 1∶400. A–D. Photographs show sections of WT (A) and KO (C) cerebellum. B and D are controls (primary antibody omitted). Arrows point to the pia mater. For clarity, tissues were not counterstained. E–H. WT (E) and KO (F–H) cerebral cortex. Insets: controls (no primary antibody) showing approximately the same regions as in the larger photographs. Tissues were counterstained with Mayer's hemalum (blue). Key: gr, stratum granulare; mol, stratum moleculare; Purkinje, Purkinje cell layer; wm, white matter. Scale bar in A = 200 µm and applies to A–D; scale in E = 100 µm and applies to E–H. (TIF) [file pone.0057292.s002.tif]

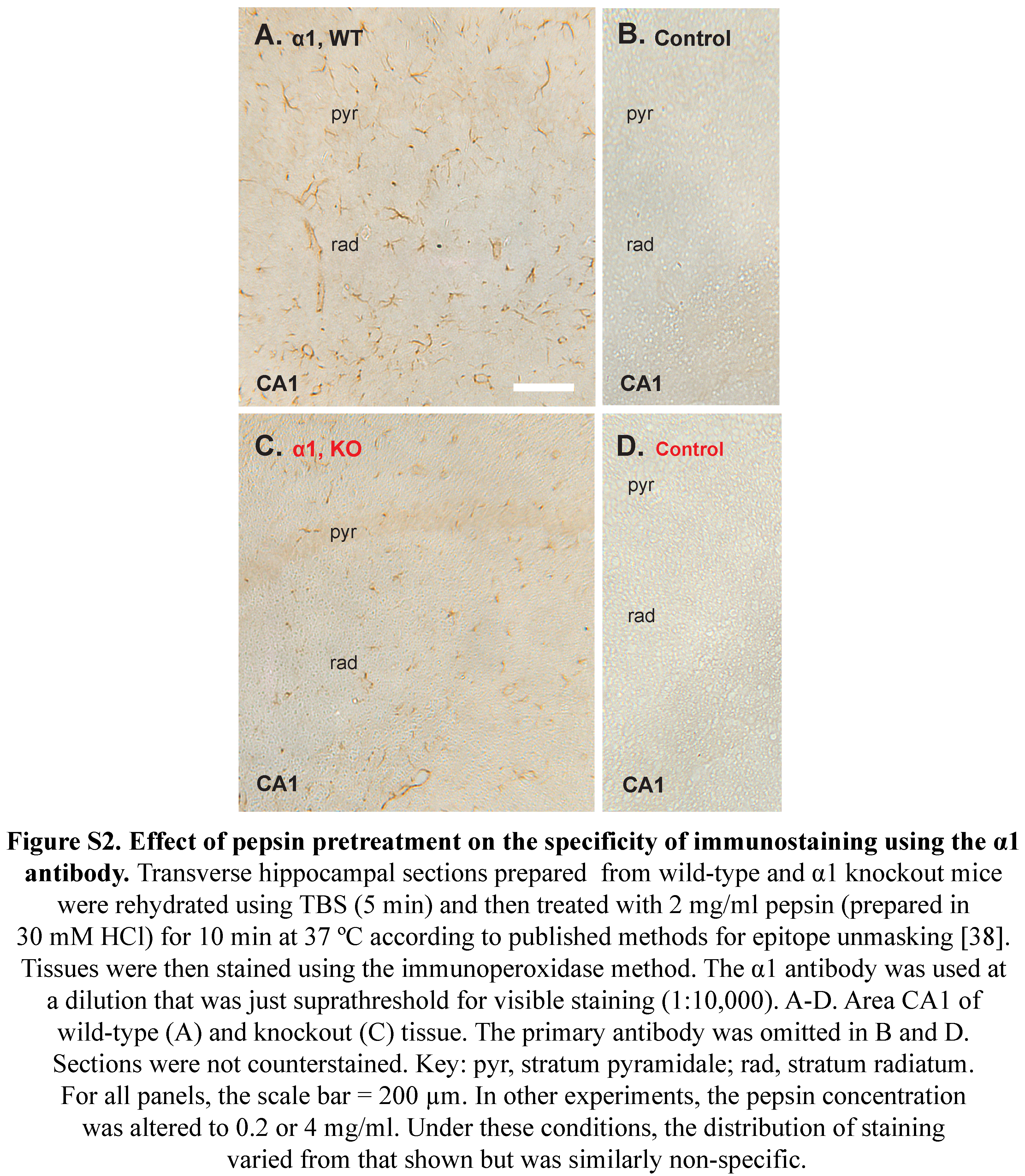

Supplement: Figure S2 — Effect of pepsin pretreatment on the specificity of immunostaining using the α1 antibody. Transverse hippocampal sections prepared from wild-type and α1 knockout mice were rehydrated using TBS (5 min) and then treated with 2 mg/ml pepsin (prepared in 30 mM HCl) for 10 min at 37°C according to published methods for epitope unmasking [38]. Tissues were then stained using the immunoperoxidase method. The α1 antibody was used at a dilution that was just suprathreshold for visible staining (1∶10,000). A–D. Area CA1 of wild-type (A) and knockout (C) tissue. The primary antibody was omitted in B and D. Sections were not counterstained. Key: pyr, stratum pyramidale; rad, stratum radiatum. For all panels, the scale bar = 200 µm. In other experiments, the pepsin concentration was altered to 0.2 or 4 mg/ml. Under these conditions, the distribution of staining varied from that shown but was similarly non-specific. (TIF) [file pone.0057292.s003.tif]

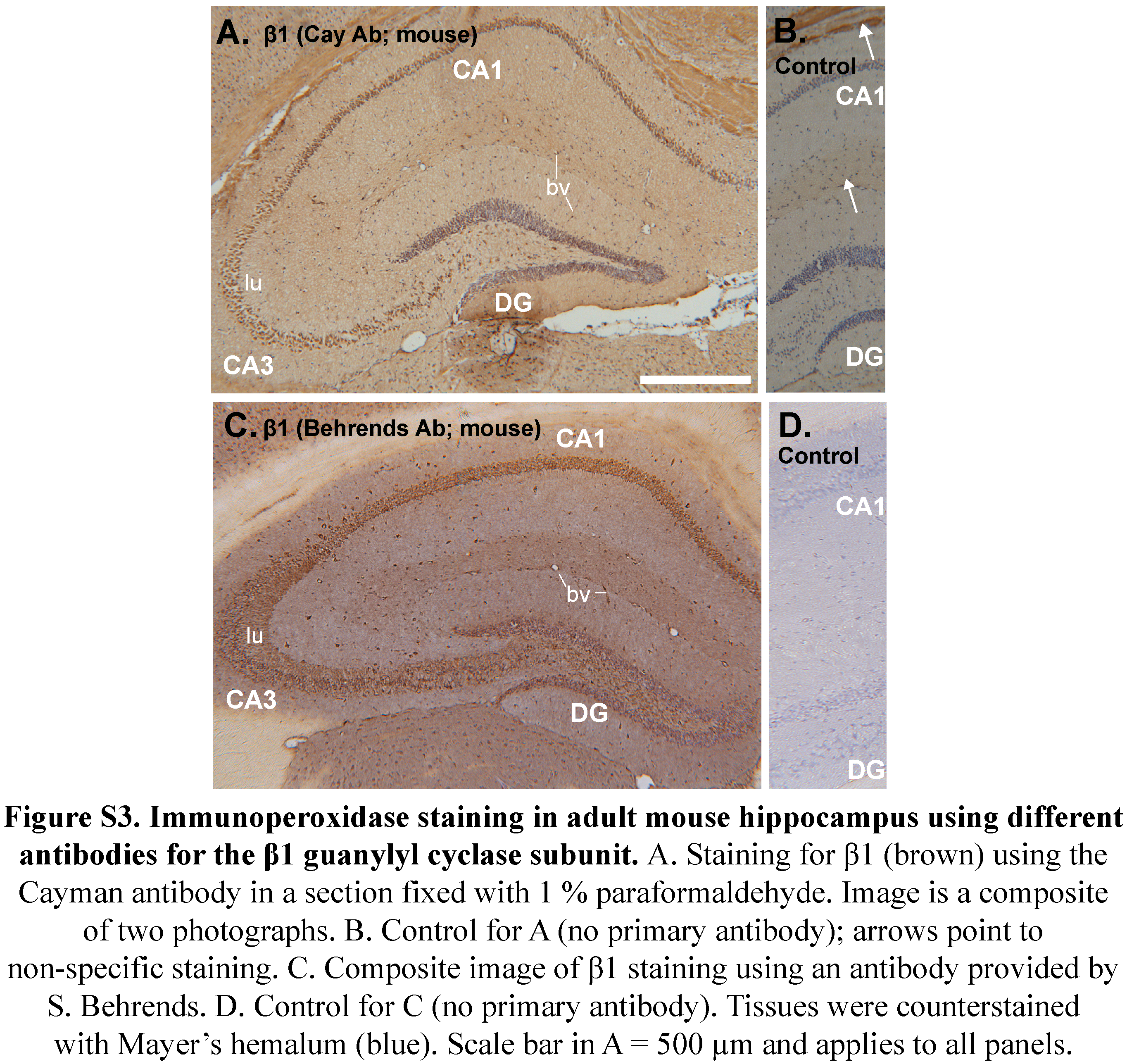

Supplement: Figure S3 — Immunoperoxidase staining in adult mouse hippocampus using different antibodies for the β1 guanylyl cyclase subunit. A. Staining for β1 (brown) using the Cayman antibody in a section fixed with 1% paraformaldehyde. Image is a composite of two photographs. B. Control for A (no primary antibody); arrows point to non-specific staining. C. Composite image of β1 staining using an antibody provided by S. Behrends. D. Control for C (no primary antibody). Tissues were counterstained with Mayer's hemalum (blue). Scale bar in A = 500 µm and applies to all panels. (TIF) [file pone.0057292.s004.tif]
